# Endo-lysosomal dysfunction in neurodegenerative diseases: opinion on current progress and future direction in the use of exosomes as biomarkers

**DOI:** 10.1098/rstb.2022.0387

**Published:** 2024-04-08

**Authors:** Mathieu Herman, Grace W. Randall, Julia L. Spiegel, Delphina J. Maldonado, Sabrina Simoes

**Affiliations:** ^1^ Taub Institute for Research on Alzheimer's Disease and the Aging Brain, Columbia University Irving Medical Center, New York, NY 10032, USA; ^2^ Department of Neurology, Columbia University Irving Medical Center, New York, NY 10032, USA

**Keywords:** endo-lysosomal system, biomarkers, neurodegenerative diseases, exosomes, endo-lysosomal dysfunction, extracellular vesicles

## Abstract

Over the past two decades, increased research has highlighted the connection between endosomal trafficking defects and neurodegeneration. The endo-lysosomal network is an important, complex cellular system specialized in the transport of proteins, lipids, and other metabolites, essential for cell homeostasis. Disruption of this pathway is linked to a wide range of neurodegenerative diseases, including Alzheimer's disease, Parkinson's disease, amyotrophic lateral sclerosis, Huntington's disease and frontotemporal dementia. Furthermore, there is strong evidence that defects in this pathway create opportunities for diagnostic and therapeutic intervention. In this *Opinion* piece, we concisely address the role of endo-lysosomal dysfunction in five neurodegenerative diseases and discuss how future research can investigate this intracellular pathway, including extracellular vesicles with a specific focus on exosomes for the identification of novel disease biomarkers.

This article is part of a discussion meeting issue ‘Understanding the endo-lysosomal network in neurodegeneration’.

## 1. The endo-lysosomal system

**Figure 1.**
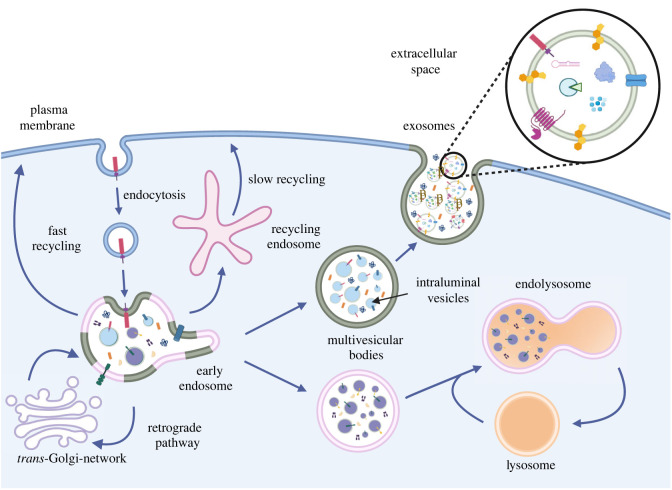
The endo-lysosomal system. Following endocytosis, cargo enters early endosomes where its fate is decided. Cargo can either be recycled back to the plasma membrane, routed to the *trans*-Golgi-network, or sorted into intraluminal vesicles (ILVs) found in multivesicular bodies (MVBs). MVBs can either fuse with lysosomes for protein degradation or with the plasma membrane. Upon fusion with the cell surface, ILVs are released into the extracellular space as exosomes. Created with BioRender.

The endo-lysosomal system consists of a dynamic network of interconnected tubulovesicular organelles, comprising early endosomes, recycling endosomes, late endosomes/multivesicular bodies and lysosomes (figure 1). This highly dynamic network plays a critical role in maintaining cell homeostasis through the control of numerous vital processes, including nutrient sensing and lipid/protein trafficking [1,2]. As depicted in figure 1, one of the main entry routes into the endo-lysosomal pathway begins with the endocytosis of a cell surface cargo and its delivery to the early endosome, where most sorting is initiated. Cargo reaching this initial sorting station can either be recycled back to the plasma membrane through the fast or slow recycling pathways, sent out to the *trans*-Golgi-network via the retrograde pathway, or sorted into the lumen of endosomes when destined for lysosomal degradation. Fate decisions between cargo degradation and cargo recycling are largely orchestrated by several endosomal machineries, including Retromer, Retriever, COMMander, and endosomal sorting complex required for transport (ESCRT) [3,4], among others. If fated for lysosomal degradation, selected cargos are sorted into intraluminal vesicles (ILVs) that progressively accumulate in the lumen of early endosomes as this compartment matures into a multivesicular body. This process is mainly mediated through ESCRT-dependent and -independent pathways that coordinate the recognition, membrane segregation, and sorting of cargos into the lumen of endosomes, as ILVs form [5,6]. Once fully matured, the endosomal compartment carrying cargos destined for degradation will fuse with a terminal storage lysosome, generating an endolysosome organelle. The combination of an acidic environment and the presence of hydrolases/proteases within this newly formed compartment facilitates the degradation of the cargo-loaded ILVs. Following cargo degradation, membranes are retrieved from the hybrid endolysosome organelle to form new lysosomes, a process called endocytic lysosome reformation [7]. Interestingly, through mechanisms currently under investigation [8], multivesicular bodies can acquire secretory capacities to directly fuse with the plasma membrane, releasing their contents into the extracellular space, including free floating (soluble) proteins and ILVs known as exosomes once secreted from the cell [9]. Exosomes belong to a large heterogeneous population of extracellular vesicles (EVs) that also include microvesicles and apoptotic bodies, among others. While some EV subtypes derive from the plasma membrane, exosomes originate from endosomes [10–13]. They are characterized as small membrane vesicles of 30–150 nm in diameter released under both normal and pathological conditions by almost any cell type in the body, including multiple cells in the brain. Exosomes play a key role in intercellular communication [10,14] and disease spread [15,16]. Since they carry a molecular signature of their parental cell, exosomes have been studied extensively as potential disease biomarkers [17]. In the following section, we will provide a brief overview, with examples, of the most relevant studies linking endo-lysosomal pathway defects and exosomal secretion to different neurodegenerative diseases.

## 2. Disruption of the endo-lysosomal system in neurodegenerative diseases

**Figure 2.**
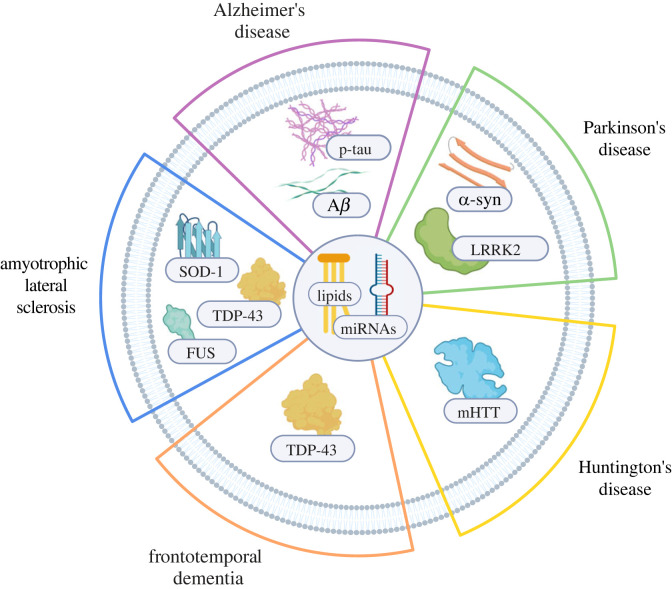
Schematic representation of key exosomal proteins found in neurodegenerative diseases. Alzheimer's disease: beta-amyloid (A*β*) and tau. Parkinson's disease: alpha-synuclein (α-syn) and leucine-rich repeat kinase 2 (LRRK2). Huntington's disease: mutant Huntington protein (mHTT). Frontotemporal dementia: TAR DNA-binding protein-43 (TDP-43). Amyotrophic lateral sclerosis: super-oxide dismutase 1 (SOD1), TAR DNA-binding protein-43 (TDP-43), and fused in sarcoma (FUS). Created with BioRender.

The endo-lysosomal system is a dynamic network whose function overlaps and interconnects with various intracellular pathways, including autophagy and phagocytosis. Impaired crosstalk between some of these pathways has been described in several diseases, but for the purpose of this *Opinion* piece, we will focus on the endo-lysosomal pathway (figure 2). Efforts have been made to investigate endo-lysosomal proteins as putative biofluid-based biomarkers. However, their low abundance in complex matrices and the use of techniques lacking sensitivity represent major obstacles. To overcome some of these challenges, targeted proteomic assays such as parallel reaction monitoring (PRM) were conducted [18]. Focusing on a predefined set of proteins, scientists detected significant changes in lysosomal enzymes and membrane proteins in biofluids from both Alzheimer's disease (AD) and Parkinson's disease (PD) patients, as well as some patients with variants of frontotemporal dementia (FTD), compared to healthy controls [19,20]; but not in Huntington's disease (HD) patients [21]. Despite such substantial advances, follow-up studies are needed to address limitations owing to cohort heterogeneity and biomarker discriminatory capacity and should include larger and longitudinal cohorts as well as patients affected by other neurodegenerative diseases. Additionally, it should be noted that targeting a specific set of proteins and excluding others may impede the discovery of novel biomarker candidates. As an alternative, exosomes derived from human biofluids, known to be enriched in the same lysosomal proteins detected in PRM studies, provide another approach to identify novel disease biomarkers.

### (a) Alzheimer's disease

AD, the most common cause of dementia worldwide, is a progressive neurodegenerative disease marked by gradual impairment of cognitive and behavioural functions [22]. Histopathologically, the disease is characterized by abnormal accumulation of extracellular beta-amyloid (A*β*) plaque deposits and intracellular tau neurofibrillary tangles in the brain [23]. One well-accepted feature of AD pathogenesis is the dysfunction of the endo-lysosomal pathway [24–26]. Endosomal trafficking defects were first discovered when an accumulation of abnormal early endosomes was observed in postmortem brain tissue from patients with AD [24,27]. In support of these findings, genetic studies revealed the existence of variants for several endosomal pathway proteins associated with elevated AD risk [28–31]. For instance, late-onset risk genes such as *BIN1*, *PICALM*, *CD2AP* and *SORL1* play various functions in endosomal trafficking [25,32–35]. Cell biology studies from our group and others demonstrated that proteins such as Retromer [36] and its associated receptor, SORL1, play a key role in the trafficking of cargo out of the endosome towards the plasma membrane [37–39], a process impaired in AD and critical for neuronal function. These discoveries reveal a promising avenue for biomarker development focused on endosomal trafficking, which we are currently investigating [40]. Exosomes have been extensively studied in the context of AD pathology, and both hallmarks of the disease—A*β* and tau species—are associated with these endosomal vesicles in mouse [41–43] and human biofluid studies [44–47]. In the last few years, substantial efforts have been made to develop AD diagnostic biomarkers using cerebrospinal fluid (CSF) and neuroimaging, and more recently blood [48]. Furthermore, the study of exosomes holds the potential not only to unravel the underlying mechanisms of AD pathogenesis and co-pathologies, but also to develop new therapeutic approaches. [49] In this context, exosomes can be used as potential vehicles for the delivery of cargo (drug, micro RNA (miRNA) and enzymes) or as ‘toxic waste scavengers' [49]. For example, a research study using an AD mouse model reported that, within the hippocampus, an intracerebral injection of glycosphingolipid-enriched exosomes can trap A*β* on their surfaces and transfer it to microglia, resulting in a reduction of A*β* pathology. This study demonstrates that exosomes can be repurposed to help with the clearance of toxic A*β* from the central nervous system [50].

### (b) Parkinson's disease

PD is a slowly progressive neurodegenerative disorder that primarily affects movement. Although the most commonly observed features of PD include tremors, impaired balance and coordination, muscle stiffness, and slow movement, the disease is also associated with a wide host of non-motor symptoms like anxiety, depression, and sleep disturbances [51]. PD is characterized by intraneuronal deposits of misfolded and aggregated alpha-synuclein (α-syn), resulting in Lewy bodies formation, and the degeneration of dopaminergic neurons in the substantia nigra pars compacta [52]. Converging lines of evidence from genetic, molecular, and model studies propose endo-lysosomal trafficking dysfunction as one of the underlying causes of PD pathophysiology [53]. Two of the intracellular defects described in PD are linked to vesicular trafficking mediated by Rab GTPase proteins [54] and defective lysosomal function [55]. Impaired acidification and degradation capacity of the lysosomal compartment result in α-syn accumulation leading to toxicity [56,57]. Concordantly, there are changes in endo-lysosomal enzyme activities in the CSF of patients with PD compared to controls [58,59]. Given the implication of endo-lysosomal dysfunction in PD, exosomal cargo has long been investigated as a source of potential biomarkers. While a 2016 study did identify phosphorylated Ser-1292 LRRK2 in urinary-derived exosomes [60], exosomal α-syn was observed in more diverse sources, including cell cultures, human CSF, plasma, and saliva [61–64]. To date, much of the literature points to α-syn species as more promising diagnostic candidates. Supporting this hypothesis, a successful cross-sectional study assessed the diagnostic performance of a CSF α-syn seed amplification assay (α-syn SAA) as able to identify individuals with PD. This assay is a major step towards therapeutic development and detection of prodromal individuals prior to diagnosis [65]. However, a notable limitation of this technology is that the test alone cannot differentiate between PD and other disorders characterized by α-syn pathology, such as multiple system atrophy and dementia with Lewy bodies. Moreover, another disadvantage is that α-syn SAA only offers a binary read-out (positive versus negative) for now, with no quantitative assessment permitting to monitor the progression of the disease. This might provide new opportunities for CSF and blood exosomes to address this gap in the field. For example, lysosomal dysfunction, a commonly accepted cytopathological feature of the disease, represents a unique sample source of exosomal biomarkers that deserves to be examined. At the same time, defects at multiple steps of the endo-lysosomal pathway are observed in conjunction with impaired crosstalks with other dysfunctional organelles, such as autophagosomes and mitochondria [57,66]. Further investigation of these interactions may unravel novel exosomal biomarkers that reflect these dysfunctions (e.g. mitochondrial and autophagic-specific cargo and proteins found in exosomes) [67,68].

### (c) Amyotrophic lateral sclerosis

Amyotrophic lateral sclerosis (ALS) is a rare neurodegenerative disease that causes gradual loss of muscle functionality. Individuals with ALS first experience muscle weakness that worsens over time, leading to paralysis and subsequent death. Symptoms result from progressive degeneration of the upper and lower motor neurons in the brain and spinal cord [69]. Genetic risk factors for ALS have aided our understanding of the cellular processes implicated in the disease's pathology. Of the 50+ genes known to be associated with the disease, nearly 20% are linked to the endo-lysosomal pathway, including endosomal maturation, lysosome biogenesis/acidification, and EV formation [69]. Interestingly, ALS is characterized by the accumulation of protein inclusions, often consisting of superoxide dismutase 1 (SOD1), TAR DNA-binding protein 43 (TDP-43), and fused-in-sarcoma (FUS), pathogenic proteins all found in exosomes isolated from CSF and plasma of ALS patients [69,70]. In addition, misfolded SOD1 participates in cell-to-cell propagation of the disease via exosomes in a prion-like manner [71], as also suggested for TDP-43 [72]. While studies have not reported differences in exosome secretion levels in ALS, there is some evidence that the mean size of plasma-derived exosomes is increased in ALS patients compared to healthy controls [73].

### (d) Huntington's disease

HD is a rare hereditary progressive neurodegenerative disease with a wide range of motor, behavioural, cognitive and psychological symptoms. Individuals with HD suffer from decline in cognitive functions, involuntary movements of the upper body, poor coordination, and psychiatric disturbances. The disease is characterized by accumulation of aggregated mutant huntingtin protein (mHTT), owing to the CAG triplet expansion in the first exon of the *HTT* gene [74]. Although a link between HD and endo-lysosomal dysfunction has been recognized in the field, it remains unclear how the endogenous and pathogenic huntingtin (HTT) proteins are related to certain aspects of this intracellular pathway, in both normal and disease state. *In vitro* and *in vivo* research using HD models suggests a role for endogenous HTT in endosome motility, tubulation, and recycling through interactions with members of the Rab family (e.g. Rabs 4, 5, 11 and HDP40). Conversely, these processes are disrupted in the presence of mHTT [75–77]. Furthermore, mHTT accumulates in endo-lysosomal compartments and can be transported in exosomes both *in vitro* and *in vivo*, suggesting that exosomes are key players in the propagation of the disease pathology [78,79]. In support of this hypothesis, a recent *in vivo* study showed elevated concentrations of both endogenous and mHTT in peripheral-derived EVs [80]. To this day, limited research has been conducted to characterize exosomes and their protein cargo in the search for HD biomarkers. Though predictive and diagnostic genetic testing is available, novel HD biomarkers identified in exosomes would be extremely useful for assessing disease progression and monitoring emerging therapies.

### (e) Frontotemporal dementia

FTD comprises a heterogeneous clinical group of neurodegenerative disorders that result from the damage leading to the loss of nerve cells in the frontal and/or temporal lobes of the brain. FTD presents with a host of progressive symptoms including behavioural and personality changes, language impairments, and in certain cases, motor neuron disturbances [81]. Unlike other forms of dementia, FTD can affect younger individuals. Both genetic and sporadic forms of the disease exist. In genetic FTD, mutations were identified in microtubule-associated protein tau (*MAPT*) [82], progranulin (*GRN*) [83,84], chromosome 9 open reading frame 72 (*C9orf72*) [85,86], and more rarely in other genes including charged multivesicular body protein 2B (*CHMP2B*) [87], *FUS* [88], and TANK-binding kinase 1 (*TBK1*) [89,90]. Histopathologically, most forms of FTD are characterized by inclusions of TDP-43 and Tau [91]. In recent years, a growing body of evidence has linked lysosomal dysfunction to the onset and development of FTD [92]. For instance, *GRN*, *CHMP2B*, and *TMEM106B* mutations impact lysosome acidification and activity [93,94]. Additionally, *C9orf72* mutations affect Rab protein activity, causing disruption of the endo-lysosomal system which leads to accumulation of protein aggregates like TDP-43 and Tau [95]. Supporting these observations, several studies demonstrated that proteins involved in FTD pathogenesis can be secreted by exosomes [72,96,97]. Consolidating these findings, other investigations using neural stem cells, autopsy brains and plasma from genetic FTD patients all reported alterations of the endo-lysosomal system and, more specifically, changes in level, size, and content of EVs/exosomes compared to control individuals [97–101]. These recent discoveries open new opportunities to investigate FTD-specific exosomal biomarkers with diagnostic and prognostic potential.

As discussed, the endo-lysosomal system is gaining recognition as an intracellular pathway that is implicated in many neurodegenerative diseases. Studies suggest that alterations in this pathway can ultimately affect exosome production, content, and secretion. Accumulating evidence in the field identified several pathological proteins contained in exosomes for each disease [17,102] (figure 2), which may open new avenues for biomarker discovery. However, the extent to which some of these markers may be used as diagnostic tools requires further replication and investigation. It is important to note that, besides proteins, additional exosomal components including lipids [103,104] and miRNA [105,106] are valuable disease biomarker candidates but not within the scope of this *Opinion* piece. If fully validated, we believe exosomal biomarkers will result in breakthroughs for clinical trials for drugs that target disease mechanisms involving the endo-lysosomal pathway. Biomarker measurements could be used as patient inclusion criteria to monitor disease progression and assess the treatment's efficacy. In addition, the endo-lysosomal pathway is often highly interconnected with other processes and organelles like autophagy [107,108] and mitochondria [66,67]. Therefore, centring future research on these intracellular interactions may uncover novel biomarkers that are not necessarily derived from exosomes.

## 3. Challenges in developing and implementing exosome biomarkers in the clinical setting

Despite significant efforts in laboratory research to investigate exosomes as potential disease biomarkers, none of the available methods allow the complete isolation, separation, and characterization of exosomes exclusively [11,12,109]. Instead, these methods enable the separation and enrichment of EV populations, including exosomes. This has led to inconsistencies and disagreements on the nomenclature used to interpret and report results across scientific studies. To address these issues, the latest guidelines released by the International Society for Extracellular Vesicles (ISEV) in 2018 (MISEV2018) recommend that EVs with uncertain subcellular origin be classified into subtypes based on their physical characteristics, biochemical composition, and cellular origin, and referred to as such. Updated recommendations are expected in the upcoming MISEV guidelines. In this section, we will briefly review the most common methods and biosample sources used for EV isolation, presenting the advantages and disadvantages of each.

### (a) Existing methods for extracellular vesicle isolation

**Figure 3.**
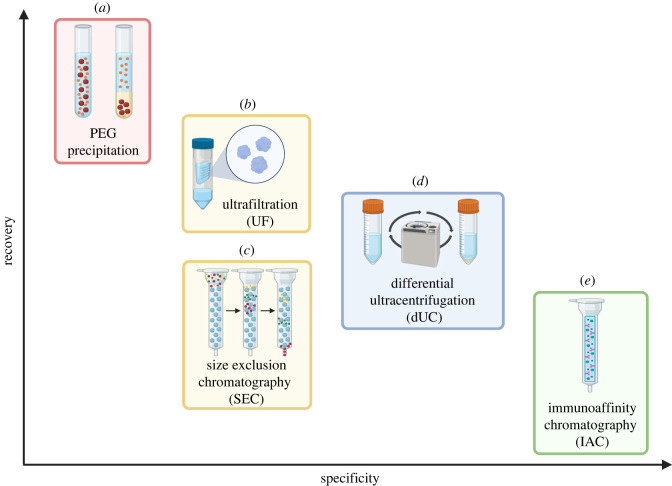
Comparison of different common EVs extraction techniques based on specificity and recovery. (*a*) polyethylene glycol (PEG) precipitation, (*b*) ultrafiltration (UF), (*c*) size exclusion chromatography (SEC), (*d*) differential ultracentrifugation (dUC), and (*e*) immunoaffinity chromatography (IAC). Created with BioRender.

**Figure 4.**
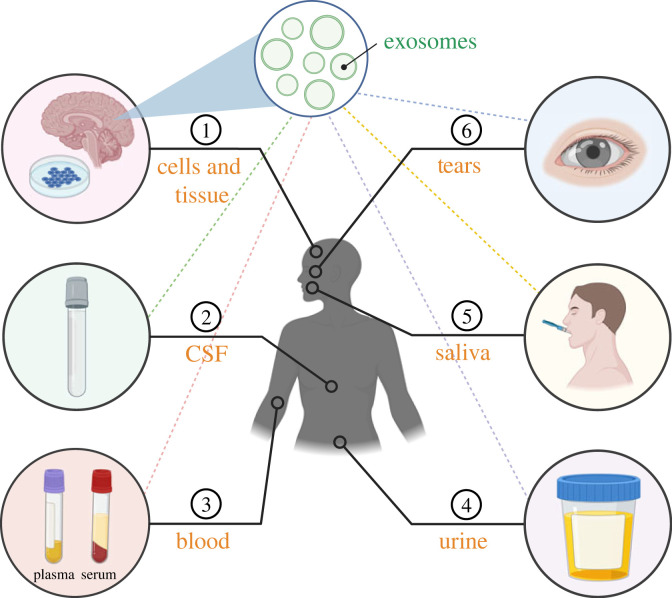
A visual representation of biosample sources for exosomes. (1) Cells and tissue, (2) cerebrospinal fluid (CSF), (3) blood, (4) urine, (5) saliva and (6) tears. Created with BioRender.

There are many protocols for isolating EVs, with new technologies emerging rapidly [110,111]. The most used methods are immunoaffinity chromatography (IAC), size exclusion chromatography (SEC), ultrafiltration (UF), differential ultracentrifugation (dUC) and polyethylene glycol (PEG) precipitation (figures 3 and 4). To date, no one-size-fits-all method exists, as each technique has its own strengths and limitations. This requires researchers to anticipate and carefully choose the most suitable method based on biosample source, downstream application, and desired purity level (specificity versus recovery) which can also be improved by combining different isolation techniques [11,12,112,113]. As one of the most effective isolation methods, IAC uses antibodies that recognize antigens displayed on the EV surface. While this technique produces an extremely pure sample, it is often unfavourable owing to its very small yield, only targeting a subset of surface markers. Compared to IAC, SEC produces a slightly larger yield with good integrity preservation. However, this technique is associated with high levels of contamination, as it isolates EVs along with similar-sized molecules like lipoproteins. Similarly, UF is prone to cross-contamination by lipoproteins, and more generally non-EV particles, and can compromise sample integrity. Yet, this technique is favourable as it is accessible, quick, easy to use, and can process a large volume of biofluid samples to produce a high yield. Another widely used method is dUC, which separates organelles based on the principle of sedimentation. Samples are sequentially spun down at different speeds, and EV-containing pellets are resuspended and can be loaded on a density gradient for further purification. Although this technique is advantageous to separate EVs from similar-sized lipoproteins, it is time-consuming. Lastly, PEG precipitation, a technique that employs a highly hydrophobic polymer, is a promising extraction method to isolate EVs from new potential biosources such as saliva and tears [64,114]. Though it is a highly efficient isolation technique requiring small volumes of biofluids, PEG precipitation is subject to a high level of contamination, making it difficult to acquire a highly purified sample. Considering these limitations, it will be crucial to implement robust and high throughput methods for EV isolation that can be applied to the research field and before implementation in the clinical setting.

### (b) Biosample sources for extracellular vesicles

Tremendous progress has been made towards the development of diagnostic biomarkers. For AD, there are Food and Drug Administration-approved diagnostic tests for the disease pathologies, amyloid plaques and tau neurofibrillary tangles, that use positron emission tomography (PET) for direct measurement of pathology or a lumbar puncture to collect CSF for measurement of A*β*42/40 or pTau181/A*β*42 [115]. The limitations of PET imaging and CSF tests are accessibility, invasiveness, and costs. These are being addressed through the use of blood-based biomarkers for AD pathology. The field is advancing towards the final steps of implementing A*β*42/40 or pTau tests globally. This involves assay standardization and generalizability of results in the real-world setting [48,116]. Despite these breakthroughs, exosomes can still offer a unique sampling strategy in search of novel biomarkers for pathologies that are not well assessed with existing biomarkers.

Exosomes (like other EV subtypes) are found in cells, tissues and biofluids. There are examples of EV isolation from many fluids: CSF, blood (plasma and serum), urine, saliva and tears. Each biofluid offers unique opportunities and challenges. As the liquid surrounding the brain and spinal cord, CSF is the most frequently used biofluid for neurodegenerative diseases [117,118]. However, CSF can only be collected by trained experts through lumbar puncture, an invasive and costly medical procedure performed under local anesthesia. In comparison, blood collection is a less invasive and widely used procedure from which serum and plasma can be separated by centrifugation. It is also a better alternative to CSF in its ability to routinely monitor and assess the progression and severity of disease. Other sources of exosomes, including urine, saliva and tears, have many advantages over blood because they do not coagulate and can be self-collected in a non-invasive way. Urine has potential to measure levels of pathological proteins [60], but it is a highly diluted fluid whose concentration may vary depending on hydration, nutrition, perspiration, kidney function, and other environmental factors. Moreover, urine is difficult to process, as urinary EV isolation requires density gradient ultracentrifugation. Compared to urine, saliva and tears are more accessible, though resources are limited. Saliva receives greater patient acceptance but is also subject to variability as well as contamination and amylase interference that can mask low-abundance proteins. Despite these challenges, a recent study reported significantly higher salivary exosome levels in patients with cognitive impairment and PD [64], encouraging further examination. Lastly, tears are of particular interest because they are secreted by the eyes, which are extensions of the brain. Promising technology has been developed to rapidly isolate tear exosomes for biomarker investigation [114]. In conclusion, the use of biofluid EVs as disease biomarkers still faces many technical obstacles before they can be implemented in the clinical setting. In fact, the pre-analytical steps including sample collection, processing, and storage, can greatly affect EV integrity, composition and downstream applications. At the same time, the complexity of each matrix owing to their biophysical and biochemical properties, has a non-negligeable effect on the purity of EV preparations. However, it is important to point out that these considerations may have limited impact on the discovery of novel EV biomarkers as long as they are highly enriched or equally distributed among EVs and non-EV particles.

## 4. Future directions

In recent years, advanced assay technology (Quanterix's Simoa ‘single molecule array’ assays, meso scale discovery biomarker assays, and Fujirebio's innotest) has been successfully developed and implemented to measure biomarker concentration levels associated with neurodegeneration. These sensitive immunoassays are widely used by the scientific community, and most assays have been optimized to process both CSF and plasma samples. While exosomes (like other subtypes of EVs) are promising candidates to discover novel biomarkers, one of the main challenges resides in improving, standardizing, and expediting isolation procedures for different subpopulations of EVs. Existing methods used in research laboratories remain time-consuming and variable among scientific teams, making exosome preparations unreliable, and impracticable clinically. It thus becomes urgent to take a step back and consider new approaches. Quanterix's Simoa digital technology offers the option to create customized ultra-sensitive immunoassays that can accurately and consistently measure biomarkers at femtomolar concentrations. Enthusiastic about this new technology, scientific groups have started using it to capture, detect and analyse EVs from diverse biosources. Customized Simoa assays using antibodies against specific exosomal transmembrane markers (tetraspanins CD9, CD63, and CD81) were developed for this purpose [119,120]. However, since tetraspanin proteins are not ubiquitously and homogeneously present on the exosomal surface, this method fails to capture all subpopulations of vesicles found in each sample, introducing possible bias in downstream biomarker analysis [121]. To overcome this issue, membrane-sensing peptides [122] that specifically recognize universal features of all small EV membranes have been developed and are being implemented in Simoa technology, by Marina Cretich and Alessandro Gori, Senior Researchers of the National Research Council of Italy at SCITEC-CNR (M. C. Cretich, A. G. Gori 2023, personal communication). This new approach could be fine-tuned for exosomal purpose, allowing their capture and analysis directly from any biofluid, without a pre-isolation step. It should also be noted that there are significant advantages to using peptides over antibodies standardly used in immunoassays. Peptides designed for assays are synthetic molecules that are cheap to produce, exhibit no lot-to-lot variability and have longer shelf life. Additionally, peptides are versatile as they can be used on different platforms. Taken together, these advances show an encouraging perspective for the future of exosome research and clinical application.

## 5. Concluding remarks

Defects in the endo-lysosomal network are implicated in disease pathogenesis, underscoring its relevance as a new source of disease biomarkers, including exosomes. As a scientific community, it is important that we reach a consensus regarding the choice of techniques for EV isolation and the use of appropriate nomenclature. Moving forward, we should support global efforts to implement standard operating procedures across laboratories to improve reproducibility, as substantial advances are made in the field. The EV-TRACK initiative, which aims to catalogue the methodology of publications that include EV-related studies, is a step in the right direction [123]. The ISEV will release their updated recommendations in the upcoming MISEV guidelines. Overall, approaching exosomes/EVs and their potential as biomarkers collectively will accelerate our understanding of neurodegenerative diseases. Furthermore, an in-depth understanding of endo-lysosomal dysfunction will potentially allow the development of new therapeutic strategies targeting this intracellular pathway to prevent, attenuate, and/or treat some of these disorders.

## Data Availability

This article has no additional data.
